# A Method for Mounting Space Telescope Optical Systems Based on the Sensitivity Matrix of Intrinsic Coefficients

**DOI:** 10.3390/s25041121

**Published:** 2025-02-12

**Authors:** Han Hou, Hongchang Ding, Keyan Dong, Guohua Cao, Boyuan Wang

**Affiliations:** 1 Changchun University of Science and Technology, No. 7089, Satellite Road, Chaoyang District, Changchun 130012, China; eric_houyz@163.com (H.H.);; 2 Chongqing Research Institute of Changchun University of Technology, No.5, Yulin Road, Yubei District, Chongqing 401120, China

**Keywords:** eigenfunctions, sensitivity matrix, space telescope, assembly methods

## Abstract

Aperture space telescopes are widely used in space debris size information detection and celestial body detection work. For the problem of limited space inside the optical system of large aperture telescopes, a space telescope mounting method based on the intrinsic coefficient sensitivity matrix is proposed by combining the wavefront detection technology. Compared with the traditional sensitivity matrix method, the method in this paper does not need to partition the detector and simplifies the construction of the wavefront reconstruction matrix. Characterisation of the system wave aberration is realised by using the eigenfactors, and the sensitivity matrix model is established according to the amount of misalignment. The experimental tests are carried out on the telescope with a diameter of 1.2 m, and the results show that the root mean square (RMS) values of the wavefront aberration in the centre field of view are less than λ/16 under the cases of eccentricity misalignment of the sub-mirror and tilting misalignment with the phase-aberration correction, which is of good value for the mounting of space telescope optical systems.

## 1. Introduction

In order to improve the detection range and detection accuracy of space telescopes, the expansion of the field of view of space telescopes is usually adopted. However, with the increasing aperture of space telescopes, higher requirements are put forward for the assembly and correction process of their optical systems [1,2]. Space telescope optical system structure is relatively complex, and usually a single optical element has six-degrees of-freedom of misalignment [3]. In the process of practical application, the optical elements after initial adjustment will also be out of adjustment due to the environment and other reasons, thus causing the imaging quality and observation accuracy of space telescope to be reduced. In order to effectively improve the imaging quality of the optical system, this paper proposes a sensitivity detection method based on the eigenfunction coefficients. Compared with the traditional Zernike polynomial curve fitting method, this method does not require partitioning of the detector and reconstruction matrix solving, and it can guarantee the fast detection of the image quality of the optical system in different observation positions, which is an effective guide for the optical system tuning of space telescopes.

Existing telescope optical systems use a sensitivity matrix method based on Zernike coefficients to achieve the characterisation of wave aberrations. In order to obtain the Zernike coefficients, wavefront reconstruction is performed by fitting a Zernike polynomial using a curvature sensor. The method requires pre-segmenting the detector and solving the wavefront reconstruction matrix. Some of the Zernike polynomials have zero curvature, and the wavefront reconstruction must be realised using the optical pupil fringe normal phase derivatives as boundary conditions [4]. The segmented edge region is used to reconstruct the Zernike polynomials with zero curvature, while further subdivision is required to improve the reconstruction accuracy. The aberration coefficients obtained by the wave aberration characterisation method based on Zernike coefficients are still different from the actual aberration coefficients and are not the optimal form of aberration characterisation. Compared with other wavefront reconstruction methods, although the direct closed-loop method can realise wavefront reconstruction of different optical pupils, it is less capable of distinguishing between curvature signals and boundary signals, and its iteration efficiency is low [5]. The direct Fourier transform method has a relatively high reconstruction speed, but its reconstruction results are prone to distortion, and the algorithm has a limited scope of application [6]. The green function method has a higher difficulty in solving the ring and square pupil, and the algorithm has a large amount of computation and poor real-time performance [7].

Aiming at the drawbacks of the traditional Zernike coefficient sensitivity matrix and related wavefront reconstruction methods, this paper proposes to use the method based on the eigenfunction to reconstruct the out-of-tune wavefront. The eigenfunction method effectively simplifies the sensitivity matrix coefficient solution model and improves the wavefront reconstruction accuracy to a certain extent by constructing the sensitivity matrix model based on the eigencoefficients and the misalignment error of the optical system. Theoretically, the sensitivity matrix method based on the eigenfunctions is applicable to most optical systems, and after obtaining the eigenfunctions with different eigenapertures, the method can realise the wavefront reconstruction of the telescope optical system and the guidance of mounting correction.

Manuel et al. proposed a novel curvature wavefront sensor based on the principle of optical differentiation, capable of simultaneously detecting wavefront curvature in the x/y direction, achieving high spatial resolution, dynamic range adjustment, and insensitivity to misalignment [8]; Tan et al. introduced a solution for WFST curvature wavefront sensing based on a convolutional neural network model with a U-Net structure, which effectively improved the efficiency and accuracy of curvature wavefront calculation compared to the sensitivity matrix method [9]; Tang et al. proposed a new misalignment correction method and used a fully connected neural network to establish a mapping relationship between misalignment and discrete orthogonal unbiased finite impulse response (UFIR) matrix features, which can effectively characterise the changes in intensity and geometric shape of the spot image, achieving high-quality imaging during the alignment and observation stages of the optical system [10]; Yu W et al. proposed an error detection technology for virtual fusion assembly method in assembly process verification analysis and effectively improved the assembly accuracy of the system by using the second-order sensitivity matrix, achieving higher solution accuracy in the system centering angle [11].

Aiming at the drawbacks of the traditional Zernike coefficient sensitivity matrix method, this paper proposes a sensitivity method based on the eigenfunction to realise the misalignment correction work of space telescope. In this paper, a field-free mirror wavefront curvature sensor is used to accomplish the information detection of the aberrant wavefront, which avoids the obstruction of the optical path of the space telescope due to the introduction of additional optics. The correlation characteristics between the intrinsic modal coefficients and the optical system misalignment are analysed, and the sensitivity matrix model based on the intrinsic coefficients is constructed to realise the effective guidance of the optical system mounting. In this paper, a coaxial triple-reflector optical system with a diameter of 1.2 m and a focal length of 1.65 is used as a test object, and the solved values are compared with the ideal values under the secondary mirror eccentricity misalignment amount $\left[-0.28,0.28\right]\;mm$ and tilt misalignment $\pm 0.2^{\circ }$. Based on the actual optical system parameters, the root mean square value of the wavefront aberration at the centre of the field of view is less than λ/16, and the error range is less than 10%, and the solution efficiency is improved by 15%, which confirms that the method has good effect on the misalignment correction of space telescopes, which confirms that this method has a good effect on space telescope misalignment correction.

## 2. Eigenfunction Solution Based on Field-Free Mirror Curvature Detection

The large aperture space telescope optical system has a compact internal structure, and when the more popular Shack–Hartmann wavefront curvature sensor is used for detection [12], the microlens array improves the light energy utilisation rate and the spot centre-of-mass coordinate detection accuracy, but it also causes the blockage of the telescope’s internal optical path. Moreover, in the actual detection process, when the zenith angle position of the telescope system changes, it is difficult to install and remove the microlens array in the test system. Similarly, there are similar limitations in the detection using tetrahedral wavefront sensors, shear interferometers, and other methods.

**Figure 1:**
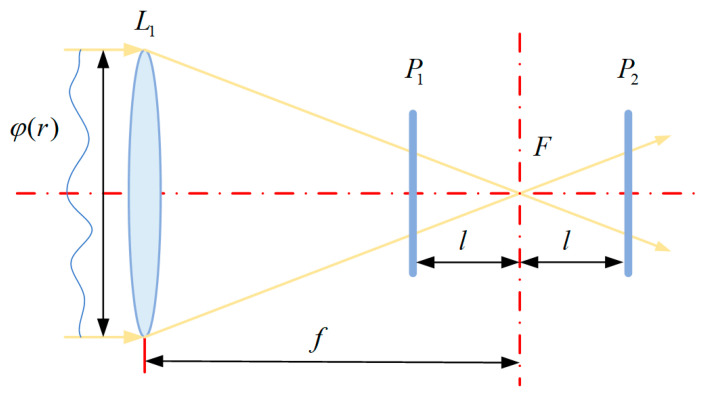
Principle of the field-free mirror wavefront curvature sensor.

To address the above drawbacks, this paper adopts a field-free mirror wavefront curvature sensor to complete the detection of wavefront information on the optical system of the telescope. The field-free mirror wavefront curvature sensor, FFCWFS, is an optical wavefront aberration sensor that provides wavefront reconstruction information by detecting the local curvature of the wavefront inside the optical pupil and the normal slope at the edge. Compared with the conventional SHWFS, the FFCWFS has a higher intrinsic sensitivity, and by directly measuring the local curvature of the wavefront, it is able to more accurately detect small changes in the wavefront, especially in the detection of higher-order aberrations [13]. The field-free mirror wavefront curvature sensor has a simple working principle, which obtains the measured wavefront information by detecting the light intensity distribution on the front and back defocused surfaces. The field-free mirror wavefront curvature sensor has the characteristics of fast reconstruction of wavefront information and the detection process without the installation of additional optical components, which can be better applied to the installation and adjustment guidance of the optical components of large aperture space telescopes with compact internal space [14]. The field-free mirror wavefront curvature sensor reduces the application of auxiliary lens at the focal point and completes the light intensity distribution at the front and back out-of-focus surfaces by a CCD detector [15], it and obtains the wavefront to be measured by solving Poisson’s equation under Neumann boundary conditions. The optical principle of the field-free mirror wavefront curvature sensor is shown in Figure 1.

As shown in Figure 1, the aberrated wavefront phase $\varphi \left(r\right)$ converges at the focal point, F, after passing through the objective lens, $L_{1}$, with focal length f. The field-free mirror wavefront curvature sensor used in this paper can achieve the direct acquisition of wavefront information without the introduction of an auxiliary lens at the focal point, F. $P_{1}$ and $P_{2}$ represent the front and back out-of-focus planes of the optical system, respectively, and $I_{1}$ and $I_{2}$ denote the intensity of the light on the front and back out-of-focus planes, and the acquired parameter information is substituted into the Poisson’s equation under Neumann’s boundary conditions to obtain the measured wavefront [16]. The measured wavefront is obtained by solving the Poisson equation under the Neumann boundary conditions.

When the incident wavefront is in an ideal state without aberration, the light intensity on the defocusing plane can be considered to be uniformly distributed. Its intensity is the same as that on the incident pupil f/l, the spot diameter is ${\left(f/l\right)}^{2}$ times the diameter of the incident pupil, and the distribution of light intensity on the defocusing plane before and after shows an inverted phenomenon. For the distorted incident light wave, the light intensity on the defocusing plane can be considered as the cumulative sum of the uniform light intensity and non-uniform light intensity in the wavefront under the ideal state under the distorted wavefront.

According to the phase transformation property of the lens, the complex amplitude of the output light wave of the lens can be expressed as Equation (1):

(1) $$A_{0}=P\left(r\right)\psi \left(r\right)exp\left(-i\pi \frac{r^{2}}{\lambda f}\right)$$

In Equation (1), r represents the spatial plane position vector of the optical system, λ is the wavelength of the light wave, $\psi \left(r\right)$ represents the complex amplitude of the incident light wave, and $P\left(r\right)$ represents the optical pupil function, with 1 and 0 representing the inner and outer pupil coefficients, respectively. Based on the Fourier transform property, after the Fresnel diffraction distance expressed as f−l, the complex amplitude on the front defocus plane, $P_{1}$, can be simplified to its integral form and expressed as $A_{1}\left(r\right)$, as shown in Equation (2).

(2) $$A_{1}\left(r\right)=\frac{1}{i\lambda \left(f-l\right)}\int P\left(r^{\prime }\right)\psi \left(r^{\prime }\right)exp\left[-i\pi \frac{r^{\prime 2}}{\lambda f}\right]exp\left[i\pi \frac{{\left(r-r^{\prime }\right)}^{2}}{\lambda \left(f-l\right)}\right]dr^{\prime }\;A_{2}\left(r\right)=P\left(r\right)\psi \left(r\right)exp\left[-i\pi \frac{r^{2}}{\lambda f}\right]*\left\{\frac{1}{i\lambda \left(f+l\right)}exp\left[i\pi \frac{r^{2}}{\lambda \left(f+l\right)}\right]\right\}\;=\frac{1}{i\lambda \left(f+l\right)}exp\left[i\pi \frac{r^{2}}{\lambda \left(f+l\right)}\right]\int P\left(r^{\prime }\right)\psi \left(r^{\prime }\right)exp\left[-i\pi \frac{lr^{\prime 2}}{\lambda \left(f+l\right)}\right]exp\left[-i2\pi \frac{rr^{\prime }}{\lambda \left(f+l\right)}\right]dr^{\prime }\;$$

In analysing the complex amplitude model of the field-free mirror wavefront curvature sensor, the front defocusing plane, $P_{1}$, of the field-free mirror wavefront curvature sensor has the same complex amplitude expression as that of the conventional curvature sensor. However, the rear defocusing plane, $P_{2}$, does not pass through the field-of-view mirror and its mathematical model can be considered as introducing a quadratic phase factor of equal size and opposite direction. Therefore, unlike conventional dual-scene wavefront curvature sensors, the complex amplitude of light waves on the back defocus plane, $P_{2}$, is expressed as $A_{2}\left(r\right)$.

Normally, the incident wave is disturbed by atmospheric turbulence, which is represented by the atmospheric coherence length, $r_{0}$. The diffraction range at the defocused plane, $P_{1}$, in front of the optical system is smaller than $\lambda \left(f-l\right)/r_{0}$. When the diffraction range of light waves is smaller than the fluctuation amplitude, the fluctuation amplitude within the $r_{0}$ range can be obtained by geometrically enlarging the range $r_{0}l/f$ at the front defocused plane, $P_{1}$. Furthermore, the defocus limit condition of the field-free mirror wavefront curvature sensor can be obtained, and its relationship is represented by Equation (3):

(3) $$\frac{\lambda \left(f-l\right)}{r_{0}}\ll \frac{r_{0}l}{f}$$

Based on the analysis of the amplitude of the light wave, it is assumed that there is only a phase change and a relatively uniform amplitude distribution in the incident wave front. In this case, the part of the spatial frequency below $1/r_{0}$ contributes less to the intensity integral function and can be considered as $\frac{l\left|\rho \right|}{\lambda f\left(f-l\right)}\leq \frac{1}{r_{0}}$ [17]. At this point $exp\left[-i\pi \frac{l\rho^{2}}{\lambda f\left(f-l\right)}\right]\approx 1$, the complex amplitude of the incident wave front is expressed as Equation (4):

(4) $$\begin{array}{c} \psi \left(r^{\prime }\right)=exp\left[i\varphi \left(r^{\prime }\right)\right]\psi \left(r^{\prime }\right)\\ \psi^{*}\left(r^{\prime }+\rho \right)=exp-i\left[\varphi \left(r^{\prime }+\rho \right)-\psi \left(r^{\prime }\right)\right] \end{array}$$

Let $r^{\prime \prime }=r^{\prime }+\rho$, when the incident wave front is a plane wave (φ=0); after Fourier transform and inverse transform processing, there is no significant change in the light intensity distribution on the $P_{1}$ surface. The light intensity on the $P_{2}$ surface can be considered to be the light intensity on the front defocused surface, $I_{1}$, obtained by defocusing transformation, i.e., l=−l. The light intensity distributions on the front and back defocused surfaces of the optical system, $I_{1}$ and $I_{2}$, are expressed as shown in Equations (5) and (6).

(5) $$I_{1}\left(r\right)=\frac{1}{\lambda^{2}{\left(f-l\right)}^{2}}\int exp\left[i2\pi \frac{\rho r}{\lambda \left(f-l\right)}\right]\int P\left(r^{\prime }\right)\left[1-i\rho \nabla \varphi \left(r^{\prime }\right)\right]exp\left[-i2\pi \frac{l\rho r^{\prime }}{\lambda f\left(f-l\right)}\right]dr^{\prime }d\rho$$

(6) $$I_{2}\left(r\right)=\frac{1}{\lambda^{2}{\left(f-l\right)}^{2}}\int exp\left[i2\pi \frac{\rho r}{\lambda \left(f-l\right)}\right]\int P\left(r^{\prime }\right)\left[1-i\rho \nabla \varphi \left(r^{\prime }\right)\right]exp\left[i2\pi \frac{l\rho r^{\prime }}{\lambda f\left(f-l\right)}\right]dr^{\prime }d\rho$$

From Equations (5) and (6), when the incident wavefront is in the ideal wavefront state without aberration, the light intensity on the defocused plane is considered to be uniformly distributed. At this time, the value of the off-focus plane light intensity is ${\left(f/l\right)}^{2}$ times at the incident pupil, the spot diameter is f/l times the pupil, and the distribution of light intensity on the front and back off-focus planes appears upside down. However, in practical application scenarios, the incident wave is usually an aberrated light wave, so the actual intensity distribution of the light wave on the front and back defocused planes is expressed in the form of $\;I\left(r\right)=I\left(r_{0}\right)+\Delta I\left(r\right)$. Equations (7) and (8) express the light intensity on the front and back defocused planes in the actual state.

(7) $$I_{1}\left(r\right)={\left(\frac{f}{l}\right)}^{2}P\left(\frac{fr}{l}\right)-\frac{\lambda f^{3}\left(f-l\right)}{2\pi l^{3}}\left[-\frac{\partial }{\partial n}\varphi \frac{fr}{l}\delta_{c}+\nabla^{2}\varphi \frac{fr}{l}\right]$$

(8) $$I_{2}\left(r\right)={\left(\frac{f}{l}\right)}^{2}P\left(-\frac{fr}{l}\right)-\frac{\lambda f^{3}\left(f+l\right)}{2\pi l^{3}}\left[-\frac{\partial }{\partial n}\varphi \left(-\frac{fr}{l}\right)\delta_{c}+\nabla^{2}\varphi \left(-\frac{fr}{l}\right)\right]$$

In Equations (7) and (8), $\delta_{c}=-\frac{\partial P}{\partial n}$ denotes the normal deviation of the edge of the optical pupil, which is infinite at the edge of the optical system aperture, and n denotes the normal vector of the edge of the optical pupil.

By analysing the light intensity distribution on the front and back defocusing planes, the field-free mirror wavefront curvature sensor can maintain the symmetry of the light intensity distribution on the front and back defocusing planes by appropriately amplifying the sampling information. By using the mathematical method to normalise the light intensity difference, the expression equation of the light intensity difference, $S\left(r\right)$, can be obtained, as shown in Equation (9) [18].

(9) $$S\left(r\right)=\left[\frac{C\cdot I_{2}\left(-r\right)-D\cdot I_{1}\left(r\right)}{C\cdot I_{2}\left(-r\right)+D\cdot I_{1}\left(r\right)}-\frac{C-D}{2D}\right]=\frac{\lambda f\left(f-l\right)}{2\pi l}\left[-\frac{\partial }{\partial n}\varphi \frac{fr}{l}\cdot \delta_{c}+\nabla^{2}\varphi \frac{fr}{l}\right]$$

In Equation (9), C and D are nonzero constants, $D/C=\left(f+l\right)/\left(f-l\right)$. r denotes the position vector in the (x,y) plane, and ϕ is the wavefront aberration of the optical pupil function. $\delta_{c}$ is infinite at the edge of the diaphragm, $\nabla_{⊥}^{2}=\frac{\partial }{\partial x^{2}}+\frac{\partial }{\partial y^{2}}$ is the transverse Laplace operator, and n represents the normal vector at the boundary of the optical pupil. Equation (9) reflects the difference in the distribution of the normalised intensity of the defocused surface before and after focusing, and it accurately reflects the change in wavefront curvature within the pupil, which is related to the normal slope of the pupil boundary. Its essence is the Poisson equation under Neumann boundary conditions, and solving Equation (9) can be used to determine the phase distribution function of the incident wave.

The above analysis proves that by adjusting the method multiplier, the field-free mirror wavefront detection method can reach the detection capability of the traditional wavefront curvature sensor. It effectively solves the limitation of not being able to add additional optical detection elements due to the limitation of the internal space structure of the telescope, and the method is applicable to most of the optical systems.

In the actual assembly process of the optical system of a space telescope, the image quality of the optical system cannot reach the ideal state due to the inherent processing errors of the optical elements. When the alignment error range is controllable, the wavefront aberration of the optical system can be expressed approximately linearly as Equation (10).

(10) $$F=F_{0j}+\frac{\partial f_{j}}{\partial x_{1}}\left(x_{1}-x_{01}\right)+\frac{\partial f_{j}}{\partial x_{2}}\left(x_{2}-x_{02}\right)+\cdots +\frac{\partial f_{j}}{\partial x_{n}}\left(x_{n}-x_{0n}\right)$$

In Equation (10), F denotes the current measured aberration value, $F_{ij}$ denotes the current residual aberration value of the optical system, and $f_{j}$ is the correspondence function of aberration to the position parameter. $\left(x_{i}-x_{oi}\right)$ is the structural displacement of the optical element, which is simplified and denoted as $\left|∆\right|x_{i}$ and includes the eccentricity, tilt, and pitch errors of the key optical elements. $\frac{\partial f_{j}}{\partial x_{n}}$ is the first-order partial derivative of the aberration to the position parameter. The approximate linear equation of wave aberration is expressed in matrix form as Equation (11).

(11) ΔF=AΔX

ΔF denotes the difference between the actual aberration and the ideal aberration; A is the sensitivity matrix determined by the design parameters of the optical system. In this study, lens processing errors, environmental disturbances, and measurement errors in the telescope optical system are ignored for a more intuitive comparison of computational efficiency. The Zernike polynomial coefficients are often used in the existing sensitivity matrix methods for aberration solution, and the eigenequations are shown in Equation (12):

(12) $$\nabla^{2}Z-{\left(x\frac{\partial }{\partial x}+y\frac{\partial }{\partial y}\right)}^{2}Z+2\left(x\frac{\partial }{\partial x}+y\frac{\partial }{\partial y}\right)+\gamma^{2}Z=0$$

From Equation (12), the Zernike polynomials are obtained by solving the unit circle eigenequations in polar coordinates.

Under the first-order approximation, the plane light intensity of $\delta_{z}$ at the distance from the focal plane can be approximated as: $I_{1/2}\approx I_{0}\left(r\right)\pm \frac{\partial I\left(r;z\right)}{\partial z}\delta z$. Based on the previous analysis of the off-focal plane light intensity distribution, the normalised light intensity difference is expressed as Equation (13):

(13) $$S\left(r\right)=\frac{I_{2}\left(-r\right)-I_{1}\left(r\right)}{I_{2}\left(-r\right)+I_{1}\left(r\right)}=-\frac{1}{I_{0}\left(r\right)}\frac{\partial I\left(r,z\right)}{\partial z}\delta_{z}=\frac{\delta_{z}}{k}\left[P\left(r\right)\nabla_{⊥}^{2}\varphi \left(r,z\right)-\frac{\partial \varphi \left(r,z\right)}{\partial n}\delta_{c}\right]$$

In Equation (13), $P\left(r\right)$ represents the pupil function. From the Fresnel diffraction theory, the solution of the square distribution at distance $\delta_{z}$ from the optical pupil can be achieved by using the normalised light intensity difference equation. When only the intensity distribution is considered, the propagation of light waves in free space follows the Helmholtz equation [19]. The light field distribution at distance $\delta_{z}$ from the optical pupil has the same form theoretically as that at the out-of-focus surface. The relationship between the optical transmission distance and the amount out of focus is expressed in Equation (14):

(14) $$\delta_{z}=\frac{f\left(f-l\right)}{l}$$

From the above analysis, the light intensity transfer function can be considered equivalent to the curvature sensor information function. Under the Neumann boundary assumption, $\gamma^{2}$ can represent the eigenvalues of the eigenfunction W(r), which organises the two-dimensional Laplace operator function as Equation (15):

(15) $$\begin{array}{c} \nabla^{2}W\left(r\right)=-\gamma^{2}W\left(r\right)\;r\in \sigma \\ \frac{\partial W\left(r\right)}{\partial n}=0\;r\in c \end{array}$$

In Equation (15), σ is the optical pupil region, c represents the boundary of the optical pupil region, and there is a mapping relationship between the eigenvalue, $\gamma_{i}$, and the eigenfunction, $W_{i}\left(r\right)$.

The Laplace operator eigenfunction in the pupil region can be solved by analysing the Neumann boundary eigenfunction under the differential chi-square condition. When defining the annular region $\left\{r_{1}\leq r\leq r_{2},0<\theta \leq 2\pi \right\}$, r denotes the polar diameter of the annular pupil, θ denotes the polar angle, and $Z=\left|m\right|,m\in 0,\pm 1,\pm 2,\cdots$, the Laplace eigenfunction in the annular pupil is expressed as Equation (16) [20].

(16) $$A_{nm}\left(r,\theta \right)=\left\{\begin{array}{ll} \left[Y_{Z}^{\prime }\left(\gamma_{nZ}r^{\prime }\right)J_{Z}\left(\gamma_{nZ}r\right)-J_{Z}^{\prime }\left(\gamma_{nZ}r^{\prime }\right)Y_{Z}\left(\gamma_{nZ}r\right)\right]cos\left(m\theta \right) & m\in [0,\infty )\\ -\left[Y_{Z}^{\prime }\left(\gamma_{nZ}r^{\prime }\right)J_{Z}\left(\gamma_{nZ}r\right)-J_{Z}^{\prime }\left(\gamma_{nZ}r^{\prime }\right)Y_{Z}\left(\gamma_{nZ}r\right)\right]sin\left(m\theta \right) & m\in \left(-\infty ,0\right) \end{array}\right.$$

In Equation (16), $J_{\mu }$ denotes the first type of Bessel function of order μ, and $Y_{\mu }$ denotes the second type of Bessel function of order μ. $\gamma_{nZ}$ is the n-th positive root of $Y_{Z}^{\prime }\left(\gamma r_{2}\right)J_{Z}\left(\gamma r_{1}\right)-J_{Z}^{\prime }\left(\gamma r_{2}\right)Y_{Z}\left(\gamma r_{1}\right)=0$. At this point $W_{k}\left(r\right)$ can be regarded as a set of orthogonal complete basis functions, then the wavefront distortion $\phi \left(r\right)$ is expressed as Equation (17):

(17) $$\varphi \left(r\right)=\sum_{k=1}^{\infty }\alpha_{k}W_{k}\left(r\right)$$

In Equation (17), $\alpha_{i}$ denotes the coefficients of the i order eigenmodes, which are used to describe the corresponding eigenmode sizes. Using the orthogonal nature of the eigenfunctions, both sides of Equation (18) are solved simultaneously to obtain the eigenfunction coefficients at different orders.

(18) $$\alpha_{k}=-c_{k}^{-1}\gamma_{k}^{-2}\int_{\sigma }W_{k}\left(r\right)\varphi \left(r\right)\nabla_{⊥}^{2}\;d\sigma$$

$c_{k}=\int_{\sigma }W_{k}^{2}\left(r\right)d\sigma$ denotes the normalisation coefficient of the function. Substituting the eigenfunction $W_{i}\left(r\right)$ and wavefront aberration $\phi \left(r\right)$ into the normalised intensity difference equation, the eigenfactor derivation equation is shown in Equation (19):

(19) $$S\left(r\right)=\frac{\lambda f\left(f-l\right)}{\pi l}\left[\nabla_{⊥}^{2}\varphi \left(\frac{f}{l}r\right)-\frac{\partial }{\partial n}\varphi \left(\frac{f}{l}r\right)\delta_{c}\right]=\frac{\lambda f\left(f-l\right)}{\pi l}\sum_{i=1}^{\infty }\left[-\alpha_{i}W_{i}\left(r\right)\gamma_{i}^{2}\right]$$

Let s=Wα; s denotes the column vectors used to describe the $S\left(r\right)$, W denotes the matrix consisting of the $-c_{0}\gamma_{i}^{2}{\;W}_{i}\left(r\right)$ column vectors, and α denotes the column vectors of the wavefront eigenfactors. After measuring the light intensity at the front and rear focal planes, the joint Equation (14) can be solved for $S\left(r\right)$, where the eigencoefficients $\alpha =W^{+}s,W^{+}$ are the generalised inverse matrix of W [21]. The wavefront aberration reconstruction is achieved by substituting α into Equation (20):

(20) $$\vec{\varphi \left(r\right)}=W_{0}\alpha =W_{0}\left(W^{+}s\right)$$

At this point, $W_{0}$ is the matrix composed of the eigenfunction $W_{i}\left(r\right)$ column vectors. Solving Equation (13) under different homogeneous Neumann boundary conditions can solve the Laplacian operator eigenfunctions in different pupil regions.

## 3. Optimisation of Sensitivity Matrix Based on Eigencoefficients

As analysed in the previous section, when the wavefront curvature detection device is combined with the traditional sensitivity matrix method for wavefront detection, the wavefront reconstruction and Zernike coefficients need to be obtained by fitting Zernike polynomials beforehand, and then the sensitivity matrix model is constructed to complete the out-of-phase solving. The above process requires pre-segmentation of the detector and reconstruction of the wavefront matrix according to its geometric distribution, which results in a relatively complex reconstruction matrix structure, a large amount of computation in the solution process, and a low reconstruction accuracy.

The Zernike coefficient sensitivity matrix has been shown to offer superior accuracy in the description of wavefront aberration due to its higher orthogonality and completeness characteristics. This method has found widespread application in off-axis reflective optical systems and Cassegrain telescope systems, where it has been employed to achieve alignment correction of optical systems through the inverse solution of misalignments, leveraging a linear relationship. However, when confronted with significant misalignment errors, the linear relationship between the Zernike coefficients and the misalignment amount becomes imbalanced, necessitating the enhancement of accuracy through the utilisation of the second-order sensitivity matrix method. This, in turn, results in an escalation in the computational complexity.

Therefore, in order to overcome the drawbacks of the Zernike coefficient sensitivity matrix, this paper proposes to use the characteristic coefficient sensitivity matrix method instead of the traditional algorithm. The intrinsic coefficient sensitivity matrix method has been shown to effectively circumvent the partition detection and reconstruction matrix calculation requirements of the traditional Zernike coefficient method, thereby facilitating rapid wavefront aberration correction. This method is particularly well-suited for the precise tuning process of optical systems with intricate internal optical path structures, and it also boasts notable advantages in optical systems with reduced overall arithmetic power or those requiring real-time, expeditious correction. In conjunction with the field-free mirror wavefront curvature sensing device proposed in this paper, it can be more effectively applied to the real-time inspection of most space telescope optical units.

Derivation of the content from Chapter 2, $\phi \left(r\right)$ is used to describe the functional mapping between wavefront aberration and the eigenfunctions of the optical system, and the mapping relationship between the optical system misalignment and the eigenfunctions is verified. The wavefront aberration measurement based on the eigenfunction method does not require detector segmentation, and the reconstruction matrix is relatively simple to solve with high efficiency. By Taylor’s theorem for multivariate functions, $f\left(x_{0}+\Delta x\right)$ can be expressed as a polynomial of order M of Δx, and the intrinsic coefficient $\alpha_{i}\;$ of the i term of the wavefront aberration can be approximated as Equation (21).

(21) $$\alpha_{i}(X+\Delta X)=\sum_{p=0}^{M}\frac{1}{p!}(\sum_{q=1}^{N}\Delta x_{q}\frac{\partial }{\partial x_{q}}{)}^{p}\cdot \alpha_{i}(X)+R_{X,M}(\Delta X)$$

X denotes the mounting degree-of-freedom covariate in the out-of-equilibrium state of the optical system; Δx is the known $q\left(1,2\ldots N\right)$ element offset; M and N denote the eigenfunction expansion order with the number of degrees of freedom, and N and $R_{X,M}\left(\Delta X\right)$ are the expansion residual.

The telescope optical system tuning process includes coarse and fine tuning. After the coarse adjustment, the system misalignment is significantly reduced and the intrinsic coefficients are linearly related to the adjustment degrees of freedom. At this point, the intrinsic coefficients $\alpha_{i}\left(x_{0}+\Delta x\right)$ are firstly expanded and expressed as Equation (22).

(22) $$\alpha_{i}\left(X+\Delta X\right)=\alpha_{i}\left(X\right)+\left(\sum_{q=1}^{N}\Delta x_{q}\frac{\partial }{\partial x_{q}}\right)\alpha_{i}\left(X\right)+R_{X,1}\left(\Delta X\right)\approx \alpha_{i}\left(X\right)+\sum_{q=1}^{N}\Delta x_{q}\frac{\partial \alpha_{i}\left(X\right)}{\partial x_{q}}$$

Assuming that the total term of the characteristic coefficients is K, the expression of the correlation matrix between the intrinsic coefficients and the assembly degrees of freedom is given by Equation (23):

(23) $$\left[\begin{array}{c} \alpha_{1}(X+\Delta X)\\ \vdots \\ \alpha_{k}(X+\Delta X)\\ \vdots \\ \alpha_{K}(X+\Delta X) \end{array}\right]=\left[\begin{array}{c} \alpha_{1}(X)\\ \vdots \\ \alpha_{k}(X)\\ \vdots \\ \alpha_{K}(X) \end{array}\right]+\left[\begin{array}{cccc} \frac{\partial \alpha_{1}(X)}{\partial x_{1}} & \frac{\partial \alpha_{1}(X)}{\partial x_{2}} & \cdots & \frac{\partial \alpha_{1}(X)}{\partial x_{N}}\\ \vdots & \vdots & & \vdots \\ \frac{\partial \alpha_{k}(X)}{\partial x_{1}} & \frac{\partial \alpha_{k}(X)}{\partial x_{2}} & \cdots & \frac{\partial \alpha_{k}(X)}{\partial x_{N}}\\ \vdots & \vdots & & \vdots \\ \frac{\partial \alpha_{K}(X)}{\partial x_{1}} & \frac{\partial \alpha_{K}(X)}{\partial x_{2}} & \cdots & \frac{\partial \alpha_{K}(X)}{\partial x_{N}} \end{array}\right]\cdot \left[\begin{array}{c} \Delta x_{1}\\ \Delta x_{2}\\ \vdots \\ \Delta x_{N} \end{array}\right]$$

Equation (23) is simplified to the form ${\alpha =\alpha }_{0}+A∆$x, where A denotes the matrix of partial derivatives of the system disorder on the eigenfactors, i.e., the eigenfactor sensitivity matrix.

From Equation (23), the optical system misalignment can be regarded as the dependent variable of the sensitivity matrix, and the intrinsic coefficient sensitivity matrix can be solved by linear fitting. In order to verify the system state in the actual mounting, the multiple mounting degrees of freedom of the optical system in the out-of-tune state are measured independently. When the eccentricity along the x axis is $D_{x}$ and the displacement range of the optical element is $\left[-0.3,0.3\right]\;mm$, the intrinsic coefficient $\alpha_{k\delta }$ at $D_{x}+\Delta D_{x\delta }$ and the intrinsic coefficient $\alpha_{k_{0}}$ in the ideal state are measured, respectively. The above measurements are fitted to a linear function as shown in Equation (24):

(24) $$\left[\begin{array}{c} \alpha_{k_{1}}-\alpha_{k_{0}}\\ \alpha_{k_{\delta }}-\alpha_{k_{0}}\\ \alpha_{k_{7}}-\alpha_{k_{0}} \end{array}\right]=\alpha \left[\begin{array}{c} \Delta D_{x_{1}}\\ \Delta D_{x_{\delta }}\\ \Delta D_{x_{7}} \end{array}\right]$$

In Equation (24), α represents the sensitivity matrix term of the intrinsic mode coefficients, which is used to describe the sensitivity of the system degrees of freedom to the optical aberration in the out-of-tune state. After completing the process of solving the sensitivity matrix of the eigenmode coefficients, the out-of-phase solution can be achieved by bringing back the intrinsic coefficients in the out-of-phase state and the intrinsic coefficients in the ideal state. Ideal intrinsic coefficient can be obtained by optical software, which can obtain the light intensity distribution and thus calculate the intrinsic function; when the high-precision optical system is used as a reference, the measurement of the light intensity distribution of the out-of-focus surface can be completed by the detector, and the intrinsic coefficient obtained at this time can be regarded as the ideal value.

**Figure 2:**
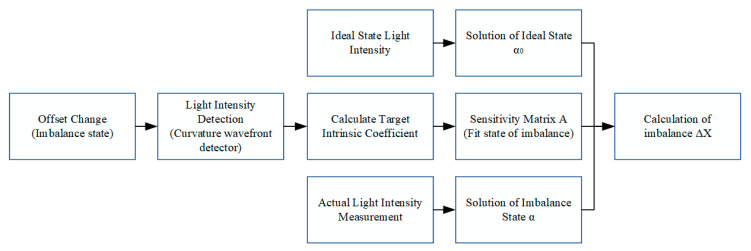
Flowchart for solving the misalignment of the intrinsic coefficient sensitivity matrix.

In the actual loading process, only a finite number of eigenvalues can be selected for wavefront reconstruction, so further selection of the evaluation coefficients is required. The eigencoefficient sensitivity matrix misalignment volume solving process is shown in Figure 2.

Analysed in the previous section, the following should be noted in the selection of the eigencoefficients in the case of a small amount of optical system misalignment: in order to improve the accuracy of the sensitivity matrix, the selected eigencoefficients should be insensitive to changes in the amount of misalignment, avoiding errors caused by the small changes in the measurement process. Due to the linear correlation between the intrinsic coefficients and the misalignment of the optical system, the fluctuation of all the mounting degrees of freedom in the optical system can be more obviously expressed when the fitting error of the sensitivity matrix term is reduced, thus improving the accuracy of the system misalignment solving.

In order to improve the accuracy of the sensitivity matrix of the eigenfunction coefficients, this paper selects the ring spot detection collector for information sampling and reconstructs the eigenfunction data using the Laplace operator. In the process of solving the wavefront function, the sample set of the eigenfunctions is limited, and it is necessary to further optimise the ring-domain eigenfunction ordering method and complete the simulation analysis [22]. From the previous section, some of the eigenfunctions do not change with the amount of system misalignment under smaller fluctuation conditions, so this paper only lists the trends of the eigenmode coefficients that are significantly affected by the misalignment, and the corresponding mode numbers are shown in Table 1.

**Figure 3:**
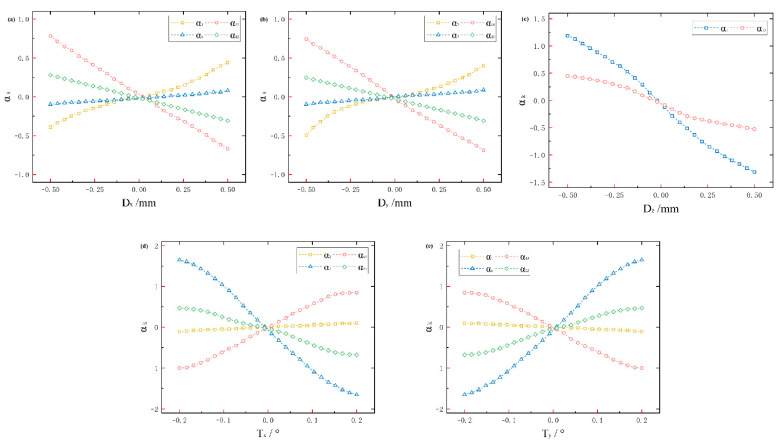
Correlation between the amount of telescope misalignment and the eigenmode coefficients.

In the coaxial three-mirror optical system selected in this paper, $D_{x},\;D_{y},\;D_{z}$ denote the eccentricity offset of the optics and $T_{x},\;T_{y},\;T_{z}$ denote the lateral tilt offset of the optics. $D_{x},\;T_{y}$ mainly affect $\alpha_{1},\;\alpha_{6},\;\alpha_{13},\;\alpha_{22}$, $D_{y}$, $T_{x}$ mainly affect $\alpha_{2},\;\alpha_{7},\;\alpha_{14},\;\alpha_{23}$, and $D_{z}$ mainly affects $\alpha_{8},\;\alpha_{24}$. According to the test results, the relationship between the intrinsic mode coefficients and the amount of system misalignment can be approximated as linear correlation when the system misalignment is small, and the degree of linear correlation is negatively correlated with the amount of system misalignment. In this paper, the eigenmode coefficients are selected from Table 1 to construct the sensitivity matrix model, and the linear results are shown in Figure 3.

From the previous analysis, the bias matrix of the eigenmode coefficients against the system misalignment is the eigenmode coefficient sensitivity matrix, and the optical system misalignment can be regarded as the dependent variable of the sensitivity matrix. Saturation measurements were performed on the five-degrees-of-freedom in the disordered state, and the sensitivity matrix of the disorder to the eigenmode coefficients was calculated using the fitting method. Table 2 demonstrates the sensitivity matrix of the eigenmode coefficients of the optical system under ideal conditions.

In Table 2, the column vectors of the sensitivity matrix represent the sensitivity of the corresponding degrees of freedom of the secondary mirrors in the telescope optical system to the image error, where the degree of influence of the degrees of freedom on the image error is positively correlated with the magnitude of the values. In the sensitivity matrix solving method, when the matrix value elements have large differences in the order of magnitude and the variation is not regular, the direct use of the matrix to solve for the out-of-phase quantities will lead to abnormal states of the matrix, which will lead to the invalidation of the solved values. In addition, because $D_{x}-T_{y},\;D_{y}-T_{x}$ affects the same intrinsic coefficients, the phenomenon of correlation between two compensators occurs, which may also lead to the failure of the solved value.

To solve the above problem, compensators with different values of column vectors in the sensitivity matrix are grouped separately. For telescope optical systems with high wavefront aberration, the compensator with higher sensitivity can be used for installation guidance. When the telescope aberration value is reduced, the compensator with lower sensitivity is used for the work.

Ideally, the optical spots at the front and rear defocused surfaces of the space telescope optical system have the same size, and the points on the front and rear defocused surfaces can be mapped through the calculation of the normalised optical intensity difference. However, during the actual mounting process, the degree of axial eccentricity between the primary and secondary mirrors of the optical system changes under the influence of axial eccentricity $D_{z}$. When the $D_{z}$ increases, the mapping points on the front and rear out-of-focus spots are separated, and the difference between the inner and outer radii increases, and then the position of the system image plane changes significantly, which leads to spot alignment errors on the out-of-focus surface, and the curvature sensor cannot be used to solve the error value, and it is necessary to re-adjust the detector to complete the focusing of the optical system.

When the curvature sensor is in an eccentric state, the misalignment of $D_{z}$ will lead to spot alignment error, which affects the calculation accuracy of the tuning degree of freedom. The effect of $D_{z}$ on the intrinsic modes is different from that of the other four degrees of freedom, and it should be a priority correction term. In the trial of this paper, the sensitivity matrix is divided into two groups for compensation: the sensitivity matrix of the compensator $A_{1}$, which includes $D_{z},\;T_{x},\;T_{y}$, and the sensitivity matrix of the compensator $A_{2\;}$, which includes $D_{x},\;D_{y}$. During the simulation process of the sensitivity matrix compensation of the $D_{z}$, the out-of-focus factor will directly affect the compensation results and, therefore, the OD size should be kept at the same size by real-time focusing and other measures.

**Figure 4:**
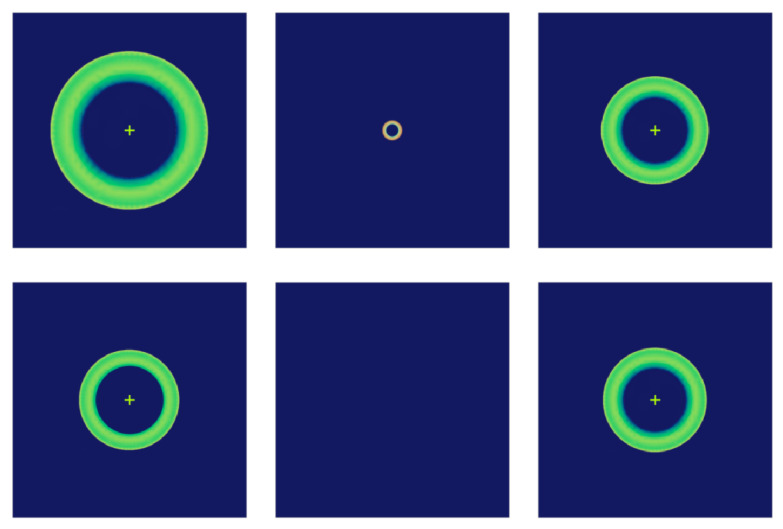
Secondary mirror axial eccentricity (no refocusing/refocusing) spot image.

**Figure 5:**
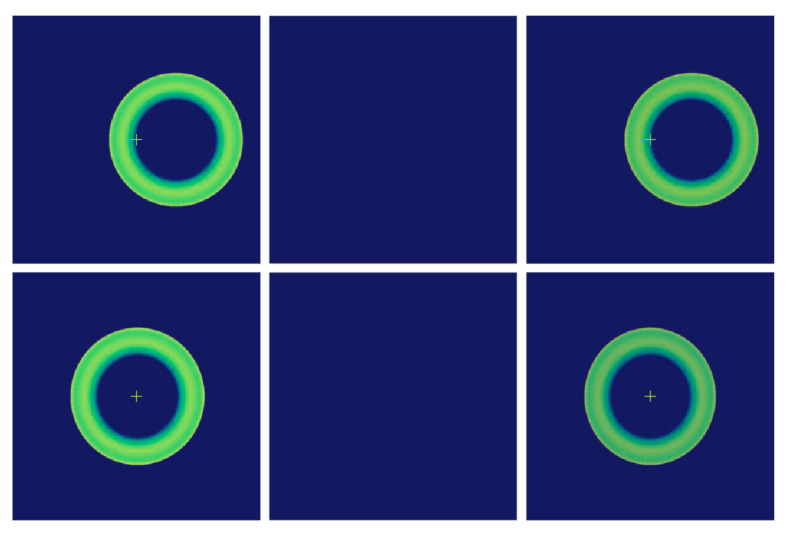
Lateral/tilted speckle image from wavefront curvature sensor.

**Figure 6:**
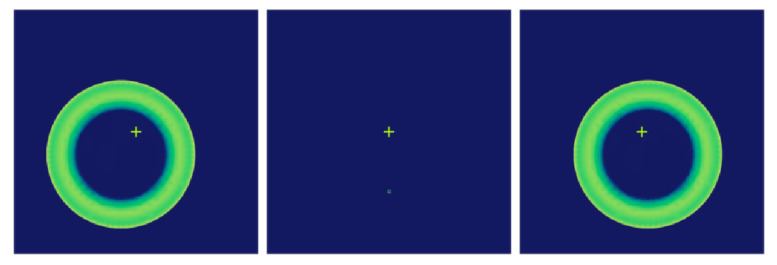
Lateral Eccentricity or Lateral Inclination Spot Images of Secondary Mirrors.

In Figure 4, the wavefront curvature sensor $D_{x}=1\;mm$ exists eccentricity, the speckle image of the wavefront curvature sensor is shifted, the speckle size remains unchanged, and the speckles on the front and rear defocus planes correspond to each other, which does not affect the calculation results. When the wavefront curvature sensor is tilted $T_{x}=1^{\circ }$, as shown in Figure 5, the spots on the out-of-focus plane maintain the mapping relationship, and at this time, it has the least impact on the calculation of the normalised intensity difference and wavefront reconstruction. When the tilt of the curvature sensor reaches a certain threshold, the spot size is significantly compressed, increasing the error in the calculation of the system’s intensity difference. When the wavefront curvature sensor is out of focus by the amount $D_{z}=-0.3\;mm$, the spot obtained on the out-of-focus plane is shown in Figure 6. The spot sizes are different and not optimally focused on the focal plane, and the focus needs to be adjusted to reduce the impact of spot size on subsequent calculations. In the actual installation and adjustment process, reducing the alignment of the wavefront curvature sensor with the primary mirror of the optical system leads to a reduction in the positional deviation of the curvature sensor, resulting in a systematic measurement process that is insensitive to the installation of the wavefront curvature sensor, which suggests that the method is applicable to the alignment correction of the optical system of a large aperture space telescope.

## 4. Experimental Setup and Adjustment of Optical System

**Figure 7:**
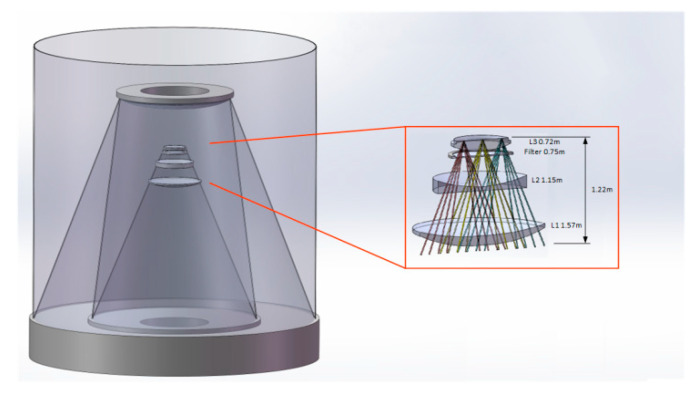
Structure of coaxial three mirror optical system.

**Figure 8:**
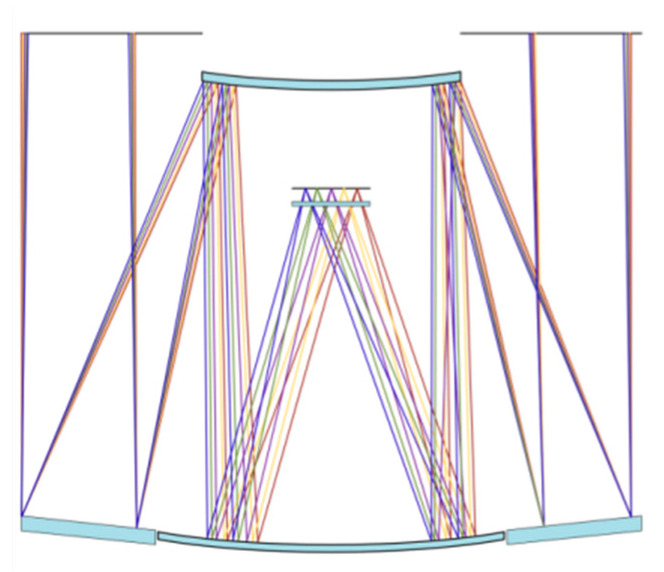
Layout of coaxial three mirror optical system.

In order to verify the effectiveness of the eigen-sensitivity matrix adjustment method proposed in this paper. As shown in Figure 7, a coaxial three-mirror optical system with an aperture of 1.2 m was used as an experimental object. The structure of the system is relatively stable, the field of view of the optical system is $3^{\circ }$, the numerical aperture of the f is 1.65, and the operating wavelength band of the system is 460~800 nm. In the optical system, the primary mirror and the third mirror are fixed to the same mirror blank, which can be regarded as keeping a relatively fixed position. The primary mirror is hyperbolic, the secondary mirror is a convex higher order asphere, and the third mirror is a concave higher order asphere. Using the primary mirror of the optical system as the adjustment reference, the adjustment parameter of the secondary mirror is considered as the offset [17]. The experiment obtains the wavefront information through a field-free mirror curvature sensor, and the detector can be scaled up appropriately to enable measurements. In order to limit the constraints and efficiency of the current experiment, the centre of the field of view is selected for the measurement experiment in this paper. According to the out-of-focus selection conditions and the requirements of measurement accuracy, the out-of-focus selection parameter is l=5 mm, the number of speckle samples is the 1/16 of the detector pixel parameter, and the detected images in the 128 pixel × 128 pixel region are selected. Figure 8 shows the layout of the 1.2 m coaxial three-mirror optical system, and Table 3 lists the main parameters of the telescope optical system.

**Figure 9:**
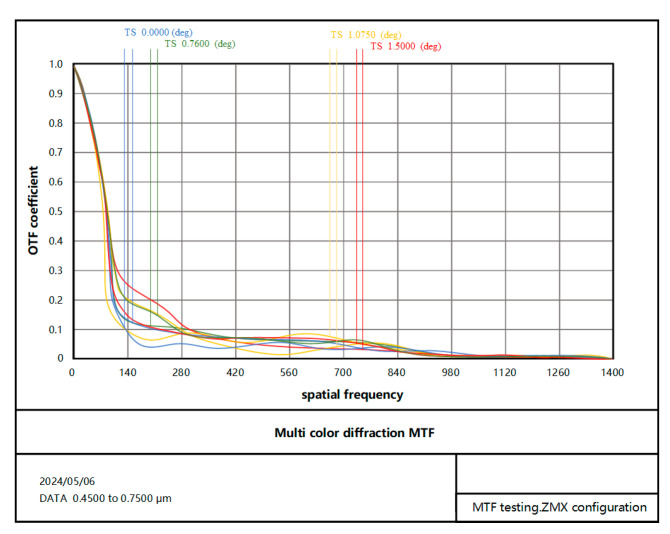
MTF diagram of the coaxial three-mirror optical system.

The testing process begins with the construction of a Zemax and Matlab dynamic data exchange communication model to control the eccentricity and tilt error of the secondary mirror of the optical system with a single variable and add wavefront aberration values layer by layer. Finally, a wavefront aberration detector is used to achieve the detection of off-focal plane light intensity values in the out-of-focus state of the optical system. In the experimental tests, the detection wavelength is chosen as 632.8 nm and the image size is 12×12 μm. The curvature sensor’s out-of-focus amount is calculated under the condition $l>>\lambda f^{2}/\lambda f+r_{0}^{2}$, and $r_{0}$ is the atmospheric coherence length. The MTF validation image of the coaxial three-mirror optical system is shown in Figure 9.

If the mounting and adjustment experiments use the primary mirror as a calibration standard, there may be an amount of error between the secondary and tertiary mirrors relative to the reference volume. Analysing the optical system structure of the space telescope, the mounting and adjustment process requires real-time detection of the secondary mirror’s six-degrees-of-freedom errors, including the translational eccentricity, $D_{x},\;D_{y},\;D_{z}$, and the angular misalignment, $T_{x},\;T_{y},\;T_{z}$. In the coaxial triple-mirror optical system, due to the rotationally symmetric nature of the system, the $T_{z}$ has virtually no effect on the system’s aberration, and, therefore, the $T_{z}$ error is not explored further, and only the secondary mirror of the system is considered contains the error with five degrees of freedom. Five groups of random misalignments are introduced into the secondary mirrors in the space telescope optical system mounting experiment, and the specific parameters are shown in Table 4.

During the testing of a coaxial triple-reflective optical system, there is mutual compensation between the transverse tilt and the eccentricity in the optical system, and at this point the compensator A1 is unable to achieve an accurate solution for the transverse eccentricity. Due to the approximate linear relationship between the intrinsic coefficients and the misalignment of the optical elements, when the misalignment error is large in magnitude, the calculation function contains a certain amount of fitting error, and relying on a single iteration can only reduce the misalignment to a certain extent and improve the linear correlation of the system. When the system contains axial eccentricity $D_{z}$, the system may introduce light spot alignment errors. When the system contains lateral eccentricity or tilt misalignment, the spot centre on the out-of-focus plane deviates from the spot centre in the ideal state, and reconstruction processing using the toroidal domain eigenfunctions will lead to image wavefront reconstruction bias. For the above situation, the compensator A1 should be used. The high sensitivity offset is calculated by the double iteration method, and by comparing the experimental data in Table 5, the system misalignment parameters after two iterations show a significant reduction trend, which further verifies the feasibility of the compensator A1.

After two adjustment iterations, the wavefront distortion detected by the compensator A1 is weakened. At this time, the compensator A2 is used to solve the alignment error, $D_{x},\;D_{y}$, of a smaller magnitude, and the calculation results are shown in Table 5.

In the process of space telescope optics assembly error correction, when a single variable such as tilt or eccentricity exists in the system, the intrinsic coefficients are affected by the amount of error to produce an approximate aberration. When the transverse eccentricity and tilted eccentricity of the secondary mirror in the optical system are calculated simultaneously, the calculation results will produce an approximate correlation effect, which will affect the accuracy of the skew calculation. In the actual installation and adjustment process, since the optical system is more sensitive to tilt error than eccentricity error, the group compensation scheme is chosen for correction in this paper. When the system wave aberration magnitude is high, the tilt error with higher sensitivity is given priority for correction; when the wave aberration value is within a reasonable threshold, the eccentricity with lower sensitivity is given priority for mounting and adjusting compensation work.

**Figure 10:**
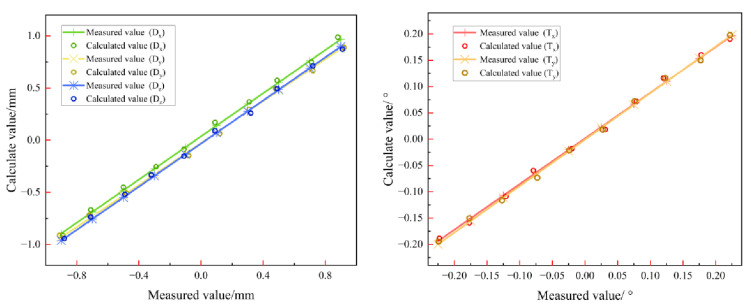
Comparison of the effect of solving single-degree-of-freedom misalignment errors.

Analysing the data in Table 5 and Table 6, in the initial state of the optical system of the telescope, the initial error of the lateral eccentricity of the system is low, and the system aberration is close to the theoretical design value after preliminary adjustment. When the initial value of transverse eccentricity is large, because there is a mutual compensation relationship between the transverse eccentricity error and the tilt error, the value of the residual tilt error after the compensator A1 is still not negligible. At this time, the residual values of $D_{x}$ and $D_{y}$ solved by the compensator *A*2 are high, so it is necessary to interpolate and compensate for the residual waveform aberration caused by the tilt error $T_{x},\;T_{y}$. Because of the sensitivity matrix of the eigenfactors, the eigenvalues converge after two iterations, and the imaging quality of the optical system is significantly improved. The validation results are shown in Figure 10.

**Figure 11:**
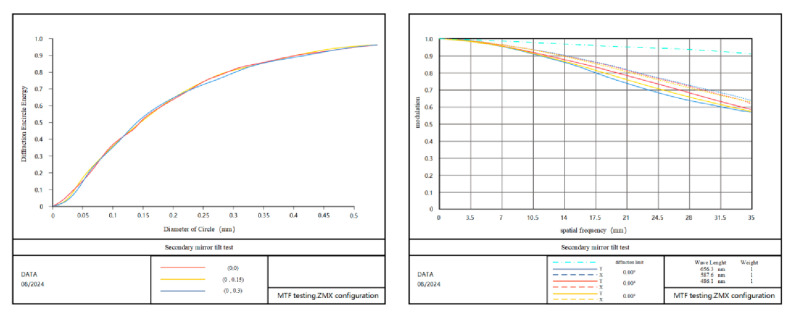
Diffraction encircled energy and MTF diagram of 1.8 m telescope secondary mirror decenter error.

**Figure 12:**
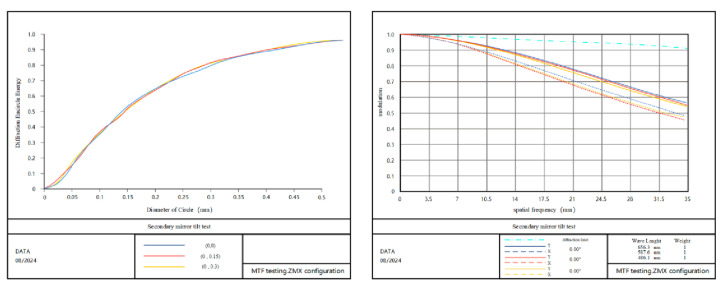
Diffraction encircled energy and MTF diagram of 1.8 m telescope secondary mirror tilt error.

In order to verify the effectiveness of the method proposed in this paper, the imaging quality of the optical system that meets the actual application environment is taken as the reference standard, and the single error amount is tested for mounting. The eccentricity measurement data $D_{x},\;D_{y}$ along the x, y, z axis and the tilt measurement data $T_{x},\;T_{y}$ along the x, y axis are obtained and compared with the theoretically calculated values. It can be concluded that the eigenvalue coefficient sensitivity matrix method can meet the calculation accuracy requirements and provide effective guidance for the state error correction of the optical system under the condition of maintaining a single adjustment degree of freedom of the optical system. Figure 11 shows the energy distribution and MTF when the secondary mirror is eccentric $D_{x}=0.07\;mm$, and Figure 12 shows the energy distribution and MTF when the secondary mirror is tilted $T_{y}=0.15{}^{\circ}$. Compared with the ideal state, the vast majority of the diffracted energy is distributed in the 2×2 image element.

Through the above experimental analysis, when the eccentricity of the secondary mirror is within the range of $\left[-0.9,0.9\right]\;mm$ and the tilt of the optical element is within the range of $\left[-0.2,0.2\right]$°, the sensitivity matrix method of the eigenfactor is used to solve and correct the misalignment error of the optical system. The results show that the method described in this paper can guide the space telescope optical system to be mounted efficiently, and the image quality of the optical system can meet the requirements of use after solving and correcting based on the eigencoefficient sensitivity matrix method, with the computational error of less than 10%, and the efficiency of solving is 15% higher than that of the traditional Zernike coefficient sensitivity matrix.

## 5. Conclusions

Aiming at the drawbacks of the traditional Zernike coefficient sensitivity matrix method and the internal space limitation of the optical system of large aperture space telescope, this paper proposes an aberration correction method of the optical system based on the intrinsic coefficient sensitivity matrix. By combining the field-free mirror wavefront curvature sensing device, the proposed method is optimised for the traditional sensitivity matrix method, without the need of zonal detection, and the reconstructed wavefront process has the features of high reconstruction accuracy, fast solution speed, and simple system structure. The optical system of the telescope with a diameter of 1.2 m and a focal length of 1.65 is experimentally verified, and the principle of group compensator is adopted to solve the solving experiments on the amount of free misalignment of a single mount. When the optical system is within the range of secondary mirror eccentricity $\left[-0.28,0.28\right]\;mm$ and tilt angle $\pm 0.2^{\circ }$, the root mean square (RMS) of the wavefront aberration at the centre of the field of view is less than λ/16, the error range is less than 10%, and the efficiency of misalignment error solving is improved by 15%. The experimental results show the good effect of the misalignment correction of the telescope optical system based on the intrinsic coefficient sensitivity matrix method. Compared with the ideal aberration of the optical system, the error of the solution results is relatively small, which is a good aid for the precision correction process of the optical system after rough adjustment and can be applied to most of the optical systems.

## Figures and Tables

**Table 1:** Eigenmode coefficient of the optical pupil.

$\alpha_{k}$	$\alpha_{1}$	$\alpha_{2}$	$\alpha_{6}$	$\alpha_{7}$	$\alpha_{8}$
m n	m = 1 n = 1	m = −1 n = 1	m = 1 n = 2	m = −1 n = 2	m = 0 n = 2
$\alpha_{k}$	$\alpha_{13}$	$\alpha_{14}$	$\alpha_{22}$	$\alpha_{23}$	$\alpha_{24}$
m n	m = 1 n = 3	m = −1 n = 3	m = 1 n = 4	m = −1 n = 4	m = 0 n = 4

**Table 2:** Parameters of the sensitivity matrix of the intrinsic mode coefficients.

$\alpha_{k}$	$D_{x}$	$D_{y}$	$D_{z}$	$T_{x}$	$T_{y}$
$\alpha_{1}$	0.476	0.003	0.061	0.357	−9.937
$\alpha_{2}$	0.013	0.514	0.043	10.027	−0.046
$\alpha_{6}$	−1.482	0	−0.039	−0.027	21.683
$\alpha_{7}$	0.013	−1.513	−0.016	−20.327	−0.044
$\alpha_{8}$	0.002	0	−3.136	0	0.049
$\alpha_{13}$	0.034	0	0	0.074	−1.21
$\alpha_{14}$	−0.009	0.054	0	1.135	−0.028
$\alpha_{22}$	−0.432	0	−0.028	−0.008	6.419
$\alpha_{23}$	0.004	−0.428	0	−6.312	−0.009
$\alpha_{24}$	0	0	−1.268	0	0.017

**Table 3:** Main parameters of the optical system of the 1.2 m space telescope.

Optical Element	Curvature Radius (mm)	Interval (mm)	Radius (mm)	Conic Coefficient	Sixth Order Non Spherical Coefficient
Mirror $L_{1}$	−2458.000	−763.730	532.473	−1.164	-
Mirror $L_{1}$	−786.630	812.230	205.326	−0.832	−3.524 × 10^−16^
Mirror $L_{1}$	−1243.265	−652.064	291.445	0.034	−1.649 × 10^−17^

**Table 4:** Amount of random error interference introduced by coaxial three-mirror telescope subscopes.

Para	$D_{x}$/mm	$D_{y}$/mm	$D_{z}$/mm	$T_{x}$/(°)	$T_{y}$/(°)
1	−0.1490	0.0840	−0.1180	−0.1370	0.1250
2	−0.1160	0.0530	0.1410	0.0960	0.1250
3	0.0370	0.0240	0.0910	−0.1650	0.1290
4	0.1360	−0.1420	−0.0087	0.1250	−0.1430
5	0.1510	−0.1370	0.0950	0.1190	−0.1380

**Table 5:** Misalignment Before and After Adjustment with A1.

Parameter	Before Alignment	After Alignment			
	Random Optical Misalignment	Rough Calculation Value	Rough Residual Error	Fine Calculation Value	Fine Residual Error
$D_{z}$/mm $T_{x}$/(°) $T_{y}$/(°)	0.1180	0.1222	−0.0042	−0.0053	0.0011
	−0.1370	−0.1228	−0.0142	−0.0133	−0.0009
	0.1250	0.1133	0.0117	0.0114	0.0003
	0.1410	0.1293	0.0117	0.0105	0.0012
	0.0960	0.0784	0.0176	0.0168	−0.0001
	0.1250	0.1160	−0.0090	−0.0112	−0.0022
	0.0910	0.0892	0.0018	−0.0009	0.0027
	−0.1650	−0.1471	−0.0179	−0.0193	0.0014
	0.1290	0.1245	0.0045	0.0124	−0.0079
	−0.0087	0.0192	0.0105	0.0093	0.0012
	0.1250	0.1145	0.0105	0.164	0.0059
	−0.1430	−0.1286	−0.0144	−0.0158	−0.0014
	0.0950	0.1463	−0.1368	−0.1924	−0.0556
	0.1190	0.1046	0.0144	0.0113	0.0031
	−0.1380	−0.1221	−0.0159	−0.0172	−0.0013

**Table 6:** Misalignment Before and After Adjustment of A2.

Parameter	Before Alignment	After Alignment			
	Random Optical Misalignment	Rough Calculation Value	Rough Residual Error	Fine Calculation Value	Fine Residual Error
$D_{x}$/mm $D_{y}$/mm	−0.1490	−0.1513	0.0023	−0.0006	0.0029
	0.0840	0.0627	0.0213	0.0195	0.0018
	−0.1160	−0.0653	−0.0507	−0.0634	0.0127
	0.0530	0.0237	0.0293	0.0342	−0.0049
	0.0370	0.0315	0.0015	0.0107	−0.0092
	0.0240	0.0148	0.0092	0.0015	0.0077
	0.1360	0.0954	0.0406	0.0143	0.263
	−0.1320	−0.1425	−0.0105	−0.0049	−0.0056
	0.1510	0.1884	−0.0374	−0.0283	−0.0091
	−0.1370	−0.1526	0.0156	0.0084	0.0072

## Data Availability

Raw data temporarily unavailable for project reasons.
